# Exploring the octanol–water partition coefficient dataset using deep learning techniques and data augmentation

**DOI:** 10.1038/s42004-021-00528-9

**Published:** 2021-06-14

**Authors:** Nadin Ulrich, Kai-Uwe Goss, Andrea Ebert

**Affiliations:** 1 Department of Analytical Environmental Chemistry, Helmholtz Centre for Environmental Research—UFZ, Leipzig, Germany; 2 Institute of Chemistry, University of Halle-Wittenberg, Halle, Germany

**Keywords:** Cheminformatics, Computational chemistry

## Abstract

Today more and more data are freely available. Based on these big datasets deep neural networks (DNNs) rapidly gain relevance in computational chemistry. Here, we explore the potential of DNNs to predict chemical properties from chemical structures. We have selected the octanol-water partition coefficient (log *P*) as an example, which plays an essential role in environmental chemistry and toxicology but also in chemical analysis. The predictive performance of the developed DNN is good with an *rmse* of 0.47 log units in the test dataset and an *rmse* of 0.33 for an external dataset from the SAMPL6 challenge. To this end, we trained the DNN using data augmentation considering all potential tautomeric forms of the chemicals. We further demonstrate how DNN models can help in the curation of the log *P* dataset by identifying potential errors, and address limitations of the dataset itself.

## Abstract (web summary)

Deep neural networks are potent tools for computational chemistry, but experimental feed data can limit their reach. Here the authors develop deep neural network data augmentation models to predict octanol–water partition coefficients (log P) of a variety of tautomers.

## Introduction

Nowadays, scientific questions in environmental sciences and toxicology are not only located in one scientific discipline like chemistry but are much more complex^1–3^. They require the combination of detailed and well-founded knowledge from a wide range of disciplines and a large number of parameters, often already in the preparation of the experiments^4,5^. These parameters, e.g., physicochemical properties of chemicals, in themselves, often require years of experience and expertise in the determination^6^. With the vast amount of chemicals in use today, it is not possible to determine experimental values for all of them. It is thus essential to have precise and profound prediction methods and models to determine these parameters reliably. Existing models for the prediction of physicochemical parameters like classical QSARs (Quantitative structure–activity relationship) are often too simplistic and limited in their application domain^7^. Chemicals that are of interest today such as ionizable chemicals, and chemicals with many functional groups were often not included in the development of the models or sometimes cannot be represented by the used substance descriptors or indices^7,8^.

With the era of networking and free availability of data, the existence of increasingly large databases, and novel techniques such as deep learning, new opportunities arise to develop improved models that can overcome existing problems and cover a wide range of chemical applications^9–11^. However, with these big datasets, new problems emerge. In such big data collections, the individual datapoints cannot be checked for plausibility manually, i.e., neither the correct mapping of the chemical structure using so-called identifiers nor the specific values of a parameter can be confirmed. It requires automated curation procedures for the preparation of datasets for model development^12^. Automation in curation of identifiers of chemical structures was enhanced strongly within the last years^13^, but the automated curation of the respective parameters or values is still pending. Manual inspections are needed, but their number can be reduced to a manageable level using error analysis, usually applying so-called ensemble models^14^. If an error is found, the value is corrected (if possible), or discarded. Simply excluding outliers without proper cause is strictly discouraged, since this would lead to overfitting and decrease the reliability of the prediction^15^. In general, the curation of experimental values is not trivial. To determine whether the value assigned to a particular chemical makes sense, or whether it is an artifact or an outlier, often requires years of experience. This includes background knowledge on the experimental methods, as well as an approximate assessment of the typical value range of different chemical structures.

Modelers and programmers, who mainly deal with the analysis of big datasets and develop deep learning models, often only have a theoretical background and lack the knowledge about the experiments behind the data and the problems regarding the experimental setups. Furthermore, scientists with in-depth backgrounds in chemistry and especially structure representation are rarely specialists in deep learning. Novel deep learning libraries like DeepChem^16,17^ help to overcome these problems. They enable a development of deep neural network (DNN) models without years of previous experience in generation of deep learning algorithms. We will demonstrate this using the octanol–water partition coefficient *K*_ow_ (in unit L_water_ L_octanol_^−1^), for which large datasets are freely available^13^. The *K*_ow_ is mostly used in its logarithmic form (log *K*_ow_) and often referred to as log *P*.

Log *P* is one of the most relevant physicochemical properties in pharmacology^18,19^, toxicology^20,21^, environmental sciences^3^, as well as in analytical chemistry^22^. It is often connected to hydrophobicity of chemicals or their lipophilicity^3^ and therefore used to quantify uptake and bioaccumulation of chemicals or drugs in organisms and specific tissues^23^ or to describe the distribution of chemicals in the environment and their sorption to sediments^24^. Log *P* is even applied to characterize chromatographic separations in reversed-phase chromatography and passive sampling devices. Although most of the processes mentioned here are more complex and can be described precisely by mechanistically based approaches, the log *P* is still the most widely applied tool. Compared to the other approaches, log *P* based descriptions are simple, and the values are readily available. Large databases such as EPI-Suite^25^, CompTox^26^, and PubChem^27^ include thousands of chemicals with experimentally determined values and offer prediction tools for chemicals without experimental data.

Experimentally determined log *P* data in research databases are already highly curated. Most of the databases refer to the data collection of Hansch et al.^28^ who documented log *P* values from different literature sources for many chemicals and selected the most trustworthy values based on many years of experience. Methods of choice for the experimental determination of the log *P* are direct methods like the shake flask method^29^, slow-stirring method^30,31^, and the generator column method^32^. For log *P* values > 5, the experimental setup is more complex. Here, the limit of detection of the respective chemical in the analysis of the two-phase system at equilibrium often becomes the limiting factor^30^. The slow-stirring method, as well as the determination by the generator column, could be used in such cases. A common indirect method for the determination of log *P* is the deduction from capacity factors of reversed-phase liquid chromatography measurements (often on a C8 or C18 column)^33,34^.

There is a large number of prediction models for log *P* values^35^. These are often performed using substructure-based methods^36^, fragmental or atom-based approaches, property-based methods^37^, or by the use of topological^38^ or E-state descriptors^39^. Another approach is the quantum-chemistry based calculation of log *P* values (e.g., the conductor-like screening model for real solvents COSMO-RS)^40^. Recently, also deep learning models to predict the log *P* have been developed. To represent the molecular input, Prasad and Brooks^41^ and Wu et al.^42^ used fingerprints and molecular descriptors to encode the molecular structure, while Wang et al. coupled canonical molecular signatures and Tree-LSTM networks to avoid countless topological features and descriptors and automate feature selection^43^.

Our goal is to establish a DNN model which allows highly accurate predictions of log *P* within a broad application domain. The model should be fast and easy to use like classical QSAR models, and perform as good as or better than any other existing prediction tool. We develop our DNN using the Python library DeepChem^17^. As molecular input we use convoluted graphs, since their use has proven very effective to predict molecular properties and activities^44^. We focus first on the preparation of the chemical dataset. We intensively work on the identification of outliers in the experimental values and demonstrate the limitations of the dataset. We test different structure representations, and demonstrate their impact on the predictive performance of the model. Further, we applied data augmentation by inclusion of all potential tautomers of a chemical to improve the predictions. The idea of data augmentation to obtain better performances when training machine learning algorithms is not new. For example, the generation of multiple conformations or the use of multiple SMILES strings for one molecule increases dataset size and can give more robust models with an improved performance^45–47^. Yet, graph convolution (as is used here) applied on multiple SMILES strings of the same molecule is likely to end up with the same graph representation. We will apply data augmentation using different tautomeric representations to overcome the problem that static graphs cannot well represent tautomers. Finally, we compare the model’s performance with seven existing prediction tools including COSMO-RS as a quantum-chemistry-based prediction tool as well as different QSAR models.

## Results

### Developed DNN models for the prediction of log *P*

**Fig. 1:**
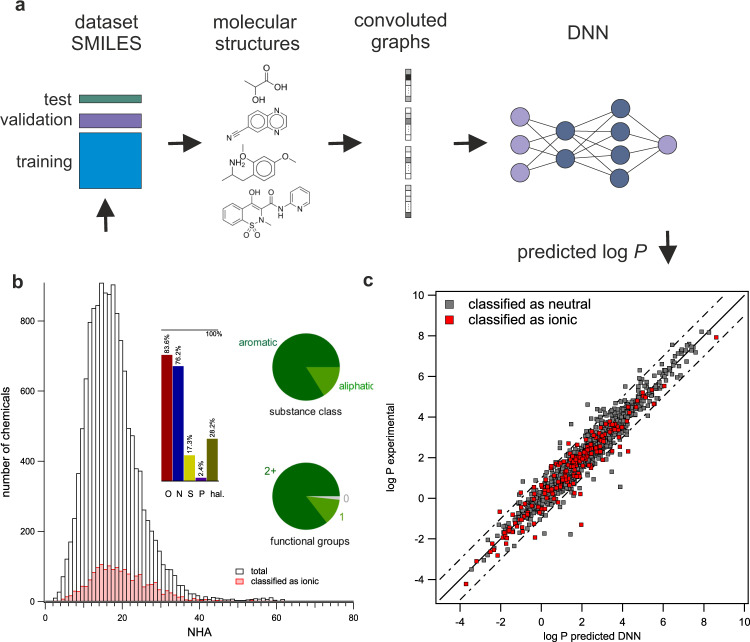
DNN development for prediction of log *P*. **a** The log *P* dataset was randomly split in training set, validation set and test set for DNN model development. The DNN is depicted schematically. **b** The dataset itself includes heterogeneous chemical structures, which can be characterized by the number of non-hydrogen atoms NHA, the number of functional groups, substance classes like aliphatic and aromatic chemicals as well as heteroatoms (O—oxygen, N—nitrogen, S—sulfur, P—phosphorous, hal.—halogens fluorine, chlorine, bromine, and iodine) included in the chemical’s structure. Further we distinguish between potential ions or neutral chemicals. **c** DNN prediction for the randomly selected test set (DNN_taut_ applied). In gray neutral chemicals are depicted, in red potential ions (anions, cations, and zwitterions) are marked.

We developed two different DNN models based on the dataset from Mansouri et al.^13^ which contains 14,050 chemicals. The dataset itself is heterogeneous and includes numerous classes of chemical compounds (Fig. 1, see Supplementary Note 1 for details). After exclusion of some identified erroneous datapoints (see *Identification of errors in the dataset*), we ended up with 13,889 chemicals. First, 10% of the dataset was randomly split as independent test data, while the remaining 90% of the data were used for model development. These remaining data (12,500 chemicals) were randomly divided into 80% training set, and 20% validation set. Different SMILES representations for each chemical (original SMILES, canonical SMILES, and SMILES with explicit Hs) as well as potential tautomers represented as SMILES codes, which were generated with JChem, were used for handling the data. Yet, ultimately the graph representation was used by the models for property prediction. For generation of the graphs as input in the first model, we only used the original form of SMILES representation, we refer to the developed model as DNN_mono_. The graphs as input for the second model were generated applying data augmentation based on all forms of SMILES and all potential tautomers, we refer to the developed model as DNN_taut_.

Best prediction performance was achieved by DNN_taut_ with a root mean square error *rmse* of 0.47 using the original SMILES representation for the test dataset (Table 1, Supplementary Table 1). The *rmse* is extremely low, compared to the experimental error that is already in the range of 0.2–0.4 log units. The DNN_mono_, which was trained on the graphs generated from the original SMILES alone had an *rmse* of 0.50 for the test dataset represented by original SMILES. But, if the chemicals of the test dataset are represented by graphs created from one of the different SMILES variants including tautomers (the respective form was selected randomly), the *rmse* for the predictions of log *P* in the test set based on the DNN_mono_ drastically deteriorates to 0.80. In contrast, the DNN_taut_ shows an unchanged *rmse* of 0.47. This means, DNN_taut_ delivers stable predictions with a high accuracy for any variant of the graph representing the chemical and each tautomeric form of the chemical.

**Table 1:** Comparison of the performance of our developed DNNs and other prediction tools.

	Test set (graphs generated from)						SAMPL6 dataset		Martel dataset	
	Original SMILES		Randomly selected^c^		Original SMILES without ions					
Model	*rmse*^d^	*sdev*	*rmse*^d^	*sdev*	*rmse*^d^	*sdev*	*rmse*^d^	*sdev*	*rmse*^d^	*sdev*
DNN_taut_^a^	0.47	±0.02	0.47	±0.02	0.45	±0.02	0.33	±0.05	1.23	0.03
DNN_mono_^a^	0.50	±0.02	0.80	±0.03	0.49	±0.02	0.31	±0.06	1.35	0.02
ACD/GALAS^b^	0.50	±0.03	0.65	±0.03	0.36	±0.02	0.51	±0.09	1.44	0.04
ALOGPS^b^	0.50	±0.02	0.66	±0.03	0.45	±0.02	0.45	±0.06	1.25	0.03
COSMO-RS^b^	0.97	±0.03	–	–	0.77	±0.03	0.37	±0.09	0.93	0.03
DataWarrior^b^	0.80	±0.02	0.92	±0.02	0.75	±0.02	0.60	±0.16	1.61	0.04
JChem^b^	0.72	±0.02	0.74	±0.03	0.69	±0.02	0.39	±0.08	1.23	0.03
KOWWIN^b^	0.65	±0.04	0.92	±0.04	0.51	±0.02	0.53	±0.09	1.38	0.04
OCHEM^b^	0.34	±0.02	0.65	±0.03	0.27	±0.02	0.49	±0.12	1.32	0.03

The root mean square error (*rmse*) and corresponding variance (*sdev*) for the log *P* prediction are given based on different SMILES inputs for the test set, the set of 11 chemicals from the SAMPL6 challenge, and the Martel dataset (707 chemicals). Results for each individual SMILES representation for the test set are given in Supplementary Table 2.

^a^Introduced in this work.

^b^Already existing prediction tool.

^c^Only one SMILES representation is randomly selected for each chemical (from the test set including also tautomers).

^d^Mean value and variance were estimated using bootstrapping. Random sampling with replacement was used to generate *N* = 1000 datasets per analyzed test set. If the *rmse* value of the original test set deviated from the calculated mean of the *rmse* distribution (*N* = 1000; one *rmse* per dataset), the mean value was reported to symmetrize the confidence intervals. The variance was determined as the standard mean error. A detailed description is given in Vorberg and Tetko^74^.

### Comparison to other prediction tools

**Fig. 2:**
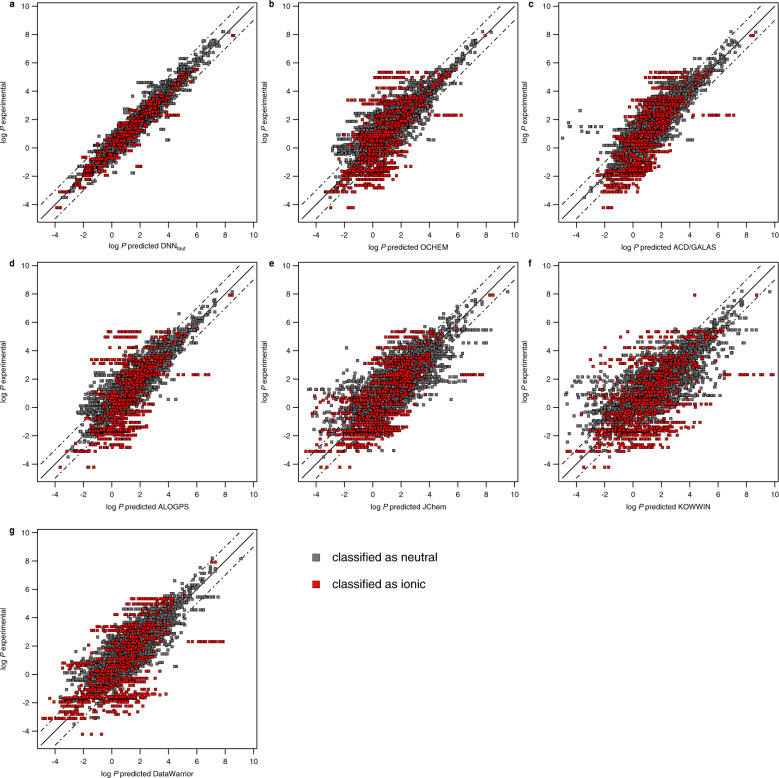
Predictions of log *P* values for the test set by our DNN_taut_ and six selected tools. The structure representation of the test set chemicals include all SMILES codes (initial, canonical, with explicit Hs) and the SMILES codes of all tautomers (multiple datapoints per chemical in case of tautomers). Neutral chemicals are marked in gray, potential ions, are marked in red. The predictions of log *P* values for the test set chemicals based on original SMILES and randomly selected SMILES (including tautomers) are shown in Supplementary Figs. 1, 2. The predictions are based on (**a**) our developed model DNN_taut_, or performed with tools from literature, namely (**b**) OCHEM, (**c**) ACD/GALAS, (**d**) ALOGPS, (**e**) JChem, (**f**) KOWWIN, and (**g**) DataWarrior.

We selected different available log *P* prediction tools for comparison: associated neural networks (OCHEM, ALOGPS), fragmental or atom-based methods (KOWWIN, ACD/GALAS, JChem, DataWarrior), and a quantum-chemistry-based calculation (COSMO-RS). Comparing the results of the DNNs with those of the other prediction tools for the test dataset (using original SMILES representations), it is noticeable that OCHEM has a significantly lower *rmse* of 0.34, while ALOGPS (*rmse* = 0.50) and ACD/GALAS (*rmse* = 0.50) show a comparable *rmse* to our DNN. The tools KOWWIN (*rmse* = 0.65), JChem (*rmse* = 0.72), DataWarrior (*rmse* = 0.80), and COSMO-RS (*rmse* = 0.97) are significantly worse in their performance (see Supplementary Fig. 1). Including tautomer representations into the test set, the *rmse* increases drastically for all tools except the DNN_taut_ (Fig. 2, COSMO-RS calculations based on the tautomer structures were not performed due to extremely time-consuming calculations). In contrast, different SMILES representations (e.g., canonical SMILES) had almost no effects. Most of the prediction tools do not explicitly consider all tautomer forms, and the dominant tautomer should be determined first to ensure precise predictions. OCHEM, the best performing model based on original SMILES as test input, shows a decrease in its predictive performance with an *rmse* of 0.65 (randomly selected SMILES variant). In case of our DNN_taut_, there is no need to determine the dominant tautomer form, which is the major advantage of our model.

### Performance of the DNN and the other tools on external datasets

We further evaluated the performance of our developed DNN_taut_ based on an external dataset, the SAMPL6 challenge^48^. Our test set is part of a freely available dataset and may have been part of the training sets of the different QSAR models. In such a case the prediction results for the test set would be misleadingly improved for these models. In contrast, the SAMPL6 dataset includes recently measured log *P* values of 11 chemicals and enables an independent comparison of all predictive tools. As can be seen from Table 1 (and Supplementary Table 3), the *rmse* of our DNN_taut_ is 0.33 which is close to the error in the experimental determination of log *P*. The performance of the DNN_mono_ is similar with an *rmse* of 0.31, because the molecules provided in the challenge had no different tautomer forms. The quantum-chemistry-based model COSMO-RS is slightly worse compared to the DNN_taut_ with an *rmse* of 0.37. Within the same range is the QSAR of JChem (*rmse* = 0.39), and also the other tools are in a range of 0.45 (ALOGPS) to 0.63 (DataWarrior), which is fairly good. Also the DNN of Prasad and Brooks^41^ performed similarly well on the SAMPL6 dataset, with an *rmse* of 0.62. Again, the DNN_taut_ is extremely stable in the performance and is as good as the quantum-chemistry based model in the prediction of log *P* but less time consuming. The SAMPL6 dataset is small; thus we added prediction results for the dataset of Martel et al.^49^ which includes log *P* values of 707 chemicals originating from the ZINC dataset (Table 1, Supplementary Note 2, Supplementary Table 4, and Supplementary Figs. 3, 4, 5). Note that all values were determined by reversed-phase liquid chromatography measurements on a C18 column. Predictions with DNN_taut_ result in an *rmse* of 1.23. The best performing model is COSMO-RS with an *rmse* of 0.93. We found two other methods in literature for the prediction of log *P*, which also included the Martel dataset. The generalized Born method in combination with solvent accessible surface area (GB/SA) method by Daina et al.^50^ reached an *rmse* of 1.56. Lui et al.^51^ used a stochastic gradient descent-optimized multilinear regression based on 1438 descriptors and reached an *rmse* of 1.03 on the Martel dataset and an *rmse* of 0.49 for SAMPL6 challenge.

### Outlier analysis based on the test set

**Fig. 3:**
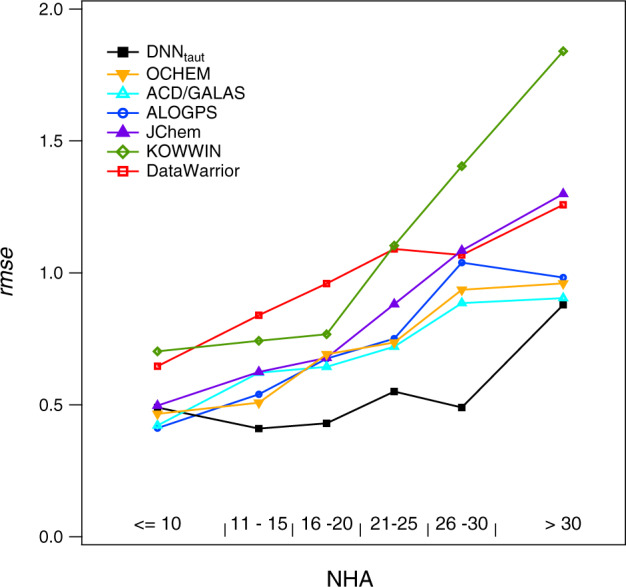
Predictions of log *P* values for the test set by our DNN_taut_ and six selected tools. The test set is represented by randomly selected SMILES codes including tautomers. The *rmse* values are shown for the range of non-hydrogen atoms NHA reflecting the size of the molecules.

Taking a closer look at the predictions of the various tools on the test set, including our developed DNNs, one can see a clear trend in the *rmse*, which is larger for chemicals with more non-hydrogen atoms (NHA). COSMO-RS, in particular, has significant problems with predictions for chemicals with greater NHA (Fig. 3, Supplementary Figs. 5, 6). Within the group of outliers (*rmse* > 1) chemicals with higher NHA are proportionally overrepresented. Such overrepresentation is also seen for ions (for categorization of ionic chemical see *Potential Limitations of the dataset: log P versus log D*), especially if they are predicted by OCHEM, COSMO-RS, and ACD/GALAS. For these models, the *rmse* significantly decreased when only chemicals classified as neutral were considered in the calculation (see Table 1). An advantage of OCHEM is a clear statement for each prediction, whether the queried chemical is within the application domain or not. Many of the ionic outliers were outside the application domain. Most of the prediction tools can in principle calculate the log *D* for the ionic form, but pH values for which the predictions are made would have to be taken into account explicitly. However, the dataset does not contain this information (see *Potential limitations of the dataset: log P versus log D*). We noted 63 compounds in the test set where at least four prediction tools showed differences greater than 1 from the experimental log *P* value, many of which were classified as ions/zwitterions or had unknown primary sources of the experimental data. We believe that all tested tools will profit from an improved classification of neutral and ionic chemicals.

### Identification of errors in the dataset

Two potential sources of error can occur for a data point in the dataset: (A) the chemical structure is not mapped correctly or (B) the corresponding value (in our case log *P*) is wrong. In case (A), false representations of the real structure are caused either by a nomenclature error in the primary source or by a wrong assignment of identifiers, such as CAS number or SMILES. The dataset used here was already curated by Mansouri et al.^13^ who checked the congruence of name, CAS, and SMILES as identifiers and added missing information like stereochemistry. For case (B), we could identify several issues in the dataset: we found mismatches in the values given in the dataset and the ones in the primary source. This is caused by transcription errors and wrong conversions (e.g., calculation of log *P* from a chromatographic capacity factor), and by the wrong assignment of experimental log *P* values for a chemical, where different log *P* values are given (e.g., Uric acid: log *P* (dataset) = −2.17 (ion), log *P* (corrected) = 0.18)^28^. Furthermore, predicted log *P* values were falsely classified as experimentally determined data. In some cases, the experimental setup was not suitable to determine the log *P* values (e.g., for Disperse Red, the solubility of the chemical limits the experimental determination of log *P* in the selected setup)^52,53^. We also identified duplicates in the dataset resulting from different structural representations of the same compound, and it is often not clear which criteria should be used to select the correct value. Tautomers can generate this problem (e.g., 4-nitrosophenol log *P* = 1.29, 4-benzoquinone mono oxime log *P* = 1.08), but also salts (e.g., octanoic acid log *P* = 3.05, sodium octanoate log *P* = −1.38, see Corrections.xlsx in GIT repository). We found eight cases in the dataset where two different representations, salt and neutral form of the chemical, are given. For these chemicals, the experimental log *P* values for the respective form are significantly different. However, no difference should occur if the corresponding pH values were selected correctly for both variants during the measurement of the log *P* value. In this case we kept the neutral form of the chemical in the dataset and excluded the salt.

To find errors and outliers in the dataset one needs to check the primary sources of the experimental data. The initial dataset included 14,050 compounds, and a manual check of all datapoints is impossible. Automation procedures for curation cannot be applied since the primary sources are hardcover books^28^, publications from the 1970 and 1980s (which are digitized by scans with low quality)^54,55^. Furthermore, some of the primary sources include also private communications, unpublished values, and primary sources which are not accessible. To identify errors in our dataset, we had to narrow down the number of values to be examined manually to a manageable number. Our strategy was developing DNN models to screen for outliers by comparing the predicted and experimental log *P* values. The hyperparameters of the DNN were optimized, several models were trained, and the results were analyzed. We defined a potential outlier as a data point where the difference between experimental log *P* and predicted log *P* in the models was greater than 1. For those outliers we checked the primary sources. In some cases, primary sources were not accessible (e.g., private communication); here, we could not correct the data point and did not exclude it from the dataset either. Using primary sources which were accessible to us, we corrected values with transcription errors and errors in the calculation of log *P* (e.g., Chlorpromazine log *P* (database) = 5.41, log *P* (corrected) = 4.00)^54^. We excluded predicted log *P* values^56,57^, and log *P* values with mismatches in the structure given in the primary source and database^55^ (see corrections.xlsx in GIT repository). We also checked if the chemical was expected to have been measured in its neutral or ionic form since we expected significant differences for ionic and zwitterionic chemicals in the prediction of log *P*.

### Potential limitations of the dataset: log *P* versus log *D*

**Fig. 4:**
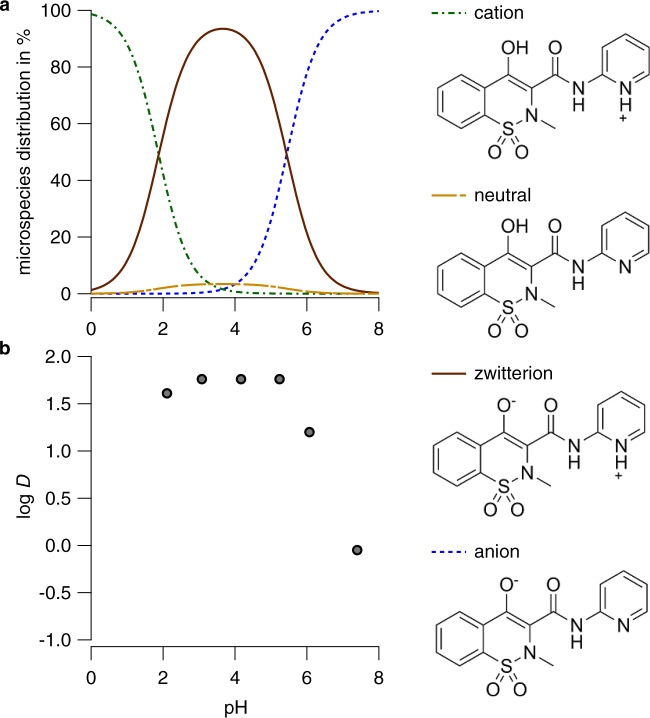
The chemical Piroxicam has four different speciations. **a** All potential speciations of Piroxicam are shown for a pH range from 0 to 8, the fractions of the ionic species dominate over the whole range of pH values. The p*K*_a_ predictions of JChem suggest that a dianion as additional species should be present at pH values > 12 (this species cannot be confirmed by p*K*_a_ measurements due to experimental limitations, the dianion is therefore not included in the plot). **b** The experimentally determined log *D* values of Piroxicam vary at different pH values. Experimental micro-p*K*_a_s and log *D* were taken from Tsai et al.

If the log *P* value is determined for ionizable chemicals, differences in the values for the neutral and ionic species are expected, and the log *P* values depend on the given pH in the experimental system. For ionizable chemicals (under consideration of the fractions *f*_species,pH_ of all possible species at a given pH value), the octanol–water partition coefficient *D* can be defined as follows: log *D* = log *∑P*_species_*f_species,pH_. An ideal model would predict log *D* instead of log *P* for a given pH value under consideration of all possible speciations of the molecule. The dataset for the development of such a model should therefore include log *D* values determined at different pH values for ionogenic chemicals (e.g., Piroxicam, Fig. 4)^58^. Hence, the model would also need to include p*K*_a_ and p*K*_b_ predictions for the chemical to determine the fractions of all ionic species. Unfortunately, the current dataset does not include information such as the pH of the system, for which the respective log *P* value was determined experimentally. Taking one step back from the ideal model by limiting the application domain, one could set the objective to precisely predict log *P* values only for neutral chemicals. Nevertheless, this is problematic since the log *P* dataset does per se not exclusively contain the log *P* values for the neutral species of an ionogenic chemical. It needs to be checked manually which experimental conditions in the log *P* determination were selected. Many primary sources do not provide this information, or while the pH of the experimental setup is defined, no reliable p*K*_a_ or p*K*_b_ values are available for the chemical.

To perform an automated check for potential ions in the dataset, we used two models from ACD (ACD/GALAS and ACD/classic) and JChem for Excel to predict p*K*_a_ and p*K*_b_ values of all chemicals. The uncertainty in the prediction of these values is quite high. Assuming precise predictions by the used tools, the same species should be predicted by all tools for a predefined pH (see Methods *Declaration of ionic chemicals in the dataset*). However, we found that this is not the case. While one tool predicts that a chemical is neutral according to our classification, another one may state that it should be present as an anion (see Dataset_and_Predictions.xlsx in GIT repository). The number of cations predicted with JChem, for example, is 817, whereas 728 are classified as cationic with ACD/GALAS. Using ACD/classic 840 chemicals are classified as cationic, of which 625 molecules show an overlap in this classification with the ACD/GALAS tool, and only 559 molecules are classified as cations by all three prediction models.

In the case of zwitterions, the total net charge is 0. However, the existing negative and positive charges in the chemical’s structure are still expected to impact the log *P* value (e.g., Piroxicam, Fig. 4)^58^. We classified a chemical as zwitterionic if its p*K*_a_ value is greater than its p*K*_b_ value. For an exact classification, micro-p*K*_a_s would be needed, which were not available. Moreover, poor performance in predicting of p*K*_a_ and p*K*_b_ can cause a wrong classification as a zwitterion or failure to recognize a zwitterion.

From these considerations, one question arises: are all ions in a prediction model outliers? As can be seen from Figs. 1, 2, this is not the case. This might in part be due to false classifications. Relatively poor predictions of p*K*_a_ and p*K*_b_ values for the chemicals included in the dataset do not ensure that all ions are identified as ions at all. Furthermore, our classification of ions was chosen in such a way that we assumed the measurement of the neutral species if it was possible to measure it at some specific pH within the range of pH 3–9. The experimental determination of the log *P* value might have been carried out under rather extreme pH values where the chemical was neutral (o-phthalic acid, measured at pH 1^28^, predicted p*K*_a_ values: 2.5 and 5.5). Alternatively, the experiment might have been carried out at a pH where the chemical was ionic, although another pH would have allowed the measurement of the neutral species (e.g., uric acid, which is neutral at pH 4, but was measured at pH 7.4 in its ionic form (log *P* = −2.92)^28^).

### Potential limitations of the dataset: structure representations for the prediction of log *P*

The structure of a chemical can be represented in many different ways. The name is one form of representation, which is often used together with the CAS registration number as an identifier. Over the years, Hansch’s original dataset^28^ has been supplemented by additional identifiers such as the SMILES code, and the plausibility of all identifiers for one chemical has been verified by various test strategies^13^. However, none of the identifiers can perfectly represent the real structure of the chemical itself. Some chemicals may, for example, have different tautomers with different stabilities (from a thermodynamic point of view) depending on the environment of the chemical^59^. For each environment of a chemical (e.g., octanol–water two-phase system) there may be different dominant tautomers. A SMILES representation of a chemical does not necessarily represent the dominant tautomer of that chemical in octanol or water. We also expect log *P* values of different tautomers of one chemical to vary^60,61^. Moreover, even if one tautomer is dominant in water or octanol, other tautomers are present in the system as well. The experimentally determined log *P* is a so-called macro log *P*, which is the sum of all micro log *P* values (of different tautomers) multiplied by the fraction *f* of each tautomer *i* present in the system: log *P*_exp_ = log ∑ *P*_i_ * *f*_i_. Even if all tautomers of a chemical can be generated from a starting SMILES representation, it remains still unclear which tautomer representation should be used in the development of the model and for predictions of log *P* values, and often an arbitrary decision for one tautomer has to be made^14^. Also, if the major tautomer is to be selected, tautomers are sometimes misrepresented in the databases^14^, and in the absence of experimental values, the dominant tautomer needs to be predicted. Yet, both experimental data and predictions of the dominant tautomer are often unsatisfactory^62^. The main advantage of our DNN_taut_ model compared to other prediction tools is that all potential tautomers (generated by JChem) of a chemical were associated with the macro log *P* value of the chemical in model training. By creating a multitude of possible tautomers for the chemicals, and adding these to our augmented training data, the resulting predictions can be based on any of the tautomer representations (test input) without a decrease in accuracy (Table 1). Nevertheless, micro log *P* values for each tautomer cannot be predicted since there is only the macro log *P* given in the dataset. But the problem that the thermodynamically favored tautomer has to be determined first to represent the structure correctly is avoided by this approach. Except for JChem, which includes the feature “consider tautomerization”, all other prediction tools decrease strongly in performance if randomly selected tautomers are used as input for the prediction. The performance of all models increases if the randomly selected tautomer is first transformed into the major tautomer using JChem, but does not reach the value with the original SMILES as test input (Table 1). COSMO-RS is not part of this comparison, since it was not possible to calculate the log *P* values for all different tautomers due to the high computing effort. Thus, COSMO-RS predictions presented here are less accurate than they could be if all tautomers were considered.

### Future perspectives for the prediction of log *P*

Analyzing the dataset and developing the models, it becomes apparent that the overall performance is extremely dependent on the quality of the dataset and necessary additional information (e.g., pH conditions of log *P* measurement). Models based on an extended dataset with log *D* values for different ionic species of a chemical would provide more precise predictions. An included, improved prediction of the p*K*_a_ and p*K*_b_ values for the chemicals would be necessary. The data augmentation applied here, which uses all possible tautomers in the training, should further be the best way to handle tautomers because the necessary log *P* values for the respective tautomer forms are not available. To conclude, DNN model development based on chemical datasets needs a lot of expertise and intensive analysis of the dataset itself even if the dataset is highly curated.

## Methods

### The log *P* dataset and generation of structure representations

Experimental log *P* values with their corresponding SMILES codes were taken from Mansouri et al.^13^. The dataset downloaded from GITHUB^63^ comprised 14,050 chemicals and resulted from the curation of the EPI-Suite dataset^25^. After curation of the initial dataset, 13,889 compounds were included. In the first step, we randomly split a test set (10%) from the dataset, the remaining dataset was split into 80% training set and 20% validation set for model development. The validation set was used for the optimization of the DNN models and to select the best DNN out of all trained DNN models. The test set was used to evaluate the overall performance of the selected DNN models (DNN_mono_ and DNN_taut_). The dataset contained three different SMILES representations: original SMILES, canonical SMILES and SMILES with explicit Hs. The original SMILES include salts and information on stereochemistry for some chemicals, the canonical SMILES (created by Indigo node in KNIME^13^) provided by Mansouri do not, yet many SMILES are identical for both sets. Marvin Suite Mol Convert for conversion from sdf file to csv file was used to extract all structure representations. In some cases problems occurred within the conversion of the original SMILES to a graph (e.g., hypervalencies in a nitro group) in DeepChem library v.2.2^16,17^, here we removed the dative bonds using OpenBabel v. 3.0.0^64^. Tautomers were generated for all SMILES representations using JChem for Excel v. 20.6.0.618^65^ (for 6212 chemicals no tautomer was received, 2849 received one tautomer, and 4828 received two or more tautomers). Duplicates in the representations were removed for DNN model development.

### Declaration of ionic chemicals in the dataset

P*K*_a_ and p*K*_b_ values were calculated for all chemicals by two models of ACD Percepta (2015 Release)—ACD/classic and ACD/GALAS, and JChem for Excel^65^. In JChem, the acidic(/basic)pKaLargeModel was used for prediction under consideration of tautomerization. It was assumed that chemicals with p*K*_a_ < 3 or p*K*_b_ > 9 were determined for a charged species. If the p*K*_a_ value was greater than the respective p*K*_b_ value, the chemical was marked as a zwitterion.

### Development of DNN models

DNN models were developed using the DeepChem library v. 2.2^16,17^ and Tensorflow v.1.14.0 in Python v. 3.5.6. SMILES of the chemicals were converted into graphs (ConvMolFeaturizer) and used as features. The feed-forward network consists of two hidden layers with 64, and 128 neurons, which were connected to a dense layer (with ReLU activation function) followed by batch normalization (including a dropout of 0.1) and the output layer (with tanh activation function). Each hidden layer was constructed by a graph convolution, a batch normalization and a graph pool layer. The DNN_mono_ and DNN_taut_ were trained for 70 and 60 epochs respectively, with a batch size of 50 and the learning rate was set to 0.00005 and 0.0001, respectively. These parameters were optimized first by evaluation of the outcomes for the validation set. Furthermore, different models (at least 3 for the complex models and 10 for the simple models) were trained. Representative training and validation curves are shown in Supplementary Fig. 7. The validation set was also used to select the final model in each case.

### Calculation of log *P* values with other software tools

Log *P*s were predicted by ALOGPS v. 2.1^66,67^, OCHEM (ochem.eu/model/4, or ALOGPS v. 3.0)^68,69^, DataWarrior v.5.2.1^70,71^, KOWWIN in EPI-SUITE^25^, JChem for Excel^65^. These predictions were made by entering respective SMILES in the software or on the website. Log *P*s computed by using the software COSMOtherm were based on the COSMO-RS (Conductor-like Screening Model for Realistic Solvation) theory and quantum chemical calculations (Turbomole and COSMOconf v. 4.1)^72,73^. The initial SMILES were used to generate COSMOfiles in COSMOconf, COSMOtherm was used with the BP_TZVPD_FINE_C30_18.ctd parametrization, at 295 K. In ACD/Percepta (2015 Release), three different prediction models—ACD/GALAS, ACD/classical, and ACD/consensus were applied for the prediction of log *P*. We only used the best performing model ACD/GALAS for comparison to other tools.

### Technical details

DNN models were trained on a Tuxedo book (Intel core i7, 64 GB RAM) using a NVIDIA RTX2080 Max Q (8 GB). The average time for training of the DNN_taut_ was ~3.5 h. COSMOtherm calculations were performed on a computing cluster system. Generation of the COSMOconf files was between 1 and 200 h for each chemical.

## Supplementary information

Supplementary Information

## Data Availability

The corrections made in the dataset (Corrections.xlsx) and the dataset used for the generation of the DNN models as well as the predicted log *P* values from all different tools (Dataset_and_Predictions.xlsx) are available at https://github.com/nadinulrich/log_P_prediction.

## Code availability

We used DeepChem library for model development. The full code for the DNN development is given at the GIT repository of DeepChem https://github.com/deepchem/deepchem. The modified code is available at https://github.com/nadinulrich/log_P_prediction.
