# Supplementary material for: Exploring the octanol–water partition coefficient dataset using deep learning techniques and data augmentation
Source: Commun Chem. 2021 Jun 14;4:90. doi: 10.1038/s42004-021-00528-9 (PMC9814212; doi:10.1038/s42004-021-00528-9)
Supplement: Supplementary file 1 — Supplementary Information [file 42004_2021_528_MOESM1_ESM.pdf]

Supplementary Information: Exploring the octanol-water partition coefficient dataset using deep learning techniques and data augmentation

Nadin Ulrich[1,*], Kai-Uwe Goss[1,2], Andrea Ebert[1]

[1]Department of Analytical Environmental Chemistry, Helmholtz Centre for Environmental Research - UFZ, Permoserstrasse 15, D-04318 Leipzig, Germany

[2]Institute of Chemistry, University of Halle-Wittenberg, Kurt-Mothes-Strasse 2, D-06120 Halle, Germany

*Corresponding Author: Phone + 49 341 235 1818 E-mail: nadin.ulrich@ufz.de

Supplementary Note 1 - Characterization of the dataset

Supplementary Note 2 – Evaluation of the models based on the external dataset of Martel et al.

Supplementary Figure 1 – Comparison of the prediction performance for the test set of our DNN_{taut} to other tools based on the original SMILES

Supplementary Figure 2 – Comparison of the prediction performance for the test set of our DNN_{taut} to other tools based on randomly selected SMILES representations (including tautomers)

Supplementary Figure 3 – Characterization of the dataset of Martel et al..

Supplementary Figure 4 – Predictions of the log P values from the dataset of Martel et al..

Supplementary Figure 5 – Influence of the number of non-hydrogen atoms NHA on the prediction performance for the test set of our DNN_{taut} to other tools based on the original SMILES

Supplementary Figure 6 – Influence of the number of non-hydrogen atoms NHA on the prediction performance for the test set of our DNN_{taut} to other tools based on the original SMILES without consideration of ionic chemicals

Supplementary Figure 7 – Rmse values of DNN_{taut} and DNN_{mono} depending on the number of epochs or training set size

Supplementary Table 1 – The different data sets can be composed of different SMILES representations (original, canonical, including explicit H atoms, tautomers), and may contain or exclude ions.

Supplementary Table 2 – Prediction results rmse of our DNN models and other prediction tools for various SMILES test sets.

Supplementary Table 3 – Prediction results of DNN_{taut} and other prediction tools for SAMPL6.

Supplementary Table 4 – Prediction results of DNN_{taut} and other prediction tools for the dataset of Martel et al..

Supplementary Note 1 - Characterization of the dataset

We investigated the dataset of Mansouri et al.[1], which contains 14,050 chemicals. After exclusion of erroneous data points (see *Identification of errors in the dataset*), 13,889 chemicals were used for model development. The dataset is heterogeneous and includes various classes of chemical compounds. 2,138 of the chemicals are classified as small molecules with an NHA (number of nonhydrogen atoms) of 1-10, 8,276 chemicals have an NHA of 11-20, 2,845 chemicals have an NHA of 21-30, and 630 chemicals are above an NHA of 30. We classified 12,076 of the chemicals as neutral, and all other chemicals are classified as possible anions, cations, or zwitterions, whereby we defined an anion by $pK_a<3$, a cation by $pK_b>9$, and a zwitterion by $pK_a<pK_b$. The dataset includes 4,092 H-bond acceptors, 60 H-bond donors, 8,781 chemicals which are H-bond acceptors as well as H-bond donors, and 949 chemicals which are classified as being neither H-bond donors nor acceptors. 16% are aliphatic, 84% are aromatic chemicals. 11,609 chemicals contain oxygen, 10,578 nitrogen, 2,403 sulfur, 336 phosphor, and 3,914 halogen atoms. 232 chemicals are carbon hydrates without heteroatoms and contain no functional groups, 1,805 chemicals contain one functional group, and 11,844 chemicals contained multiple functional groups.

Supplementary Note 2 – Evaluation of the models based on the external dataset of Martel et al.

Predictions of log *P* were further performed for the dataset of Martel et al.[2]. This dataset includes 707 chemicals (selected from ZINC collection), and log *P* values were determined by measuring retention factors on a C18 column in reversed-phase liquid chromatography. The pH values of the measurements were carefully selected to only measure neutral chemicals. The log *P* values range from 0.3 to 7, and, according to the authors, 46% of the chemicals are non-ionizable, 30% basic, 17% acidic, 0.5% zwitterionic, and 6.5% ampholytes. The majority of the chemicals contains above 25 NHA (see Supplementary Figure 3), so on average the molecules are larger than those of Mansouri's dataset (see Figure 1 for comparison). We found no chemical overlap between both datasets.

Supplementary Figure 4 shows the results for the predictions for all tools. The *rmse* values are higher compared to the test set and SAMPL6 challenge. When applying our models DNN_{taut} and DNN_{mono} on this external dataset, with an *rmse* of 1.23 and 1.35 respectively, we get a better *rmse* for our model that includes tautomers in the training set. The prediction errors are rather high for all other external tools, ranging between the best performing COSMO-RS with an *rmse* of 0.93 up to an *rmse* of 1.61 for DataWarrior. OCHEM, which performed best on the Mansouri test set, is slightly worse than our model with an *rmse* of 1.32 (see Supplementary Table 4 for all values).

For all models except COSMO-RS there seems to be a strong tendency to underestimate the log *P* values. About 50% of all extreme outliers (≥2 log units, for the example of DNN_{taut}) are large molecules with an NHA≥30, and 78% of all extreme outliers have an experimental log *P* above 4, and 40% even above 5. The bad performance of the models could also be a consequence of the lack of similar chemicals in the training set. To address this problem, we included part of the Martel dataset into our training set, in addition to the data from Mansouri.

We call these models $DNN_{taut,martel}$ if trained with data augmentation and $DNN_{mono,martel}$ if trained without data augmentation. Each model was trained two times, once with one half (randomly selected) of the chemicals from the Martel dataset included in the training set, the second time with the other half included in the training set. Each model was then used to test its performance on the remaining chemicals which were not included in the training set. The results for $DNN_{taut,martel}$ and $DNN_{mono,martel}$ are thus each a combination of two models, to cover all 707 chemicals.

The *rmse* improved for both models, with an *rmse* of 0.84 for $DNN_{mono,martel}$ and 0.89 for $DNN_{taut,martel}$. Again, the model including tautomers performs better than the one based on the original SMILES alone. After training on the dataset, it performs slightly better than COSMO-RS, which due to its *ab initio* method does not depend on a training set. In Supplementary Figure 4 b one can see that for $DNN_{taut,martel}$ the tendency to underestimate the data is not as pronounced as for DNN_{taut}, but the scatter is still quite high. While the afore mentioned high NHA numbers could be one reason, because the performance of all models tends to decrease with the number of NHA (see Figure 3), part of the variance could also be due to the experimental approach. The correlation of log *P* and a retention factor is a fast alternative method for the experimental determination of log *P*[3]. However, the OCED guideline suggests using the shake-flask method and the slow-stirring method for log P>5. There are few other standard techniques, for example, the use of a generator column. Using retention data from liquid chromatography is more erroneous compared to the other methods (where octanol and water are used as the two phases) since immobilized C18 alkyl chains at silica particles are one phase and a water - organic solvent mix is the second phase. Octanol (C8) is not the same as C18 modified silica particles, and of course, a water – organic solvent mixture (often methanol or acetonitrile are used) is not comparable to water solely. Further, the partition process in chromatography is different from a two-phase equilibrium in a flask.

Supplementary Figure 1 – Comparison of the prediction performance for the test set of our DNN$_{taut}$ to other tools based on the original SMILES

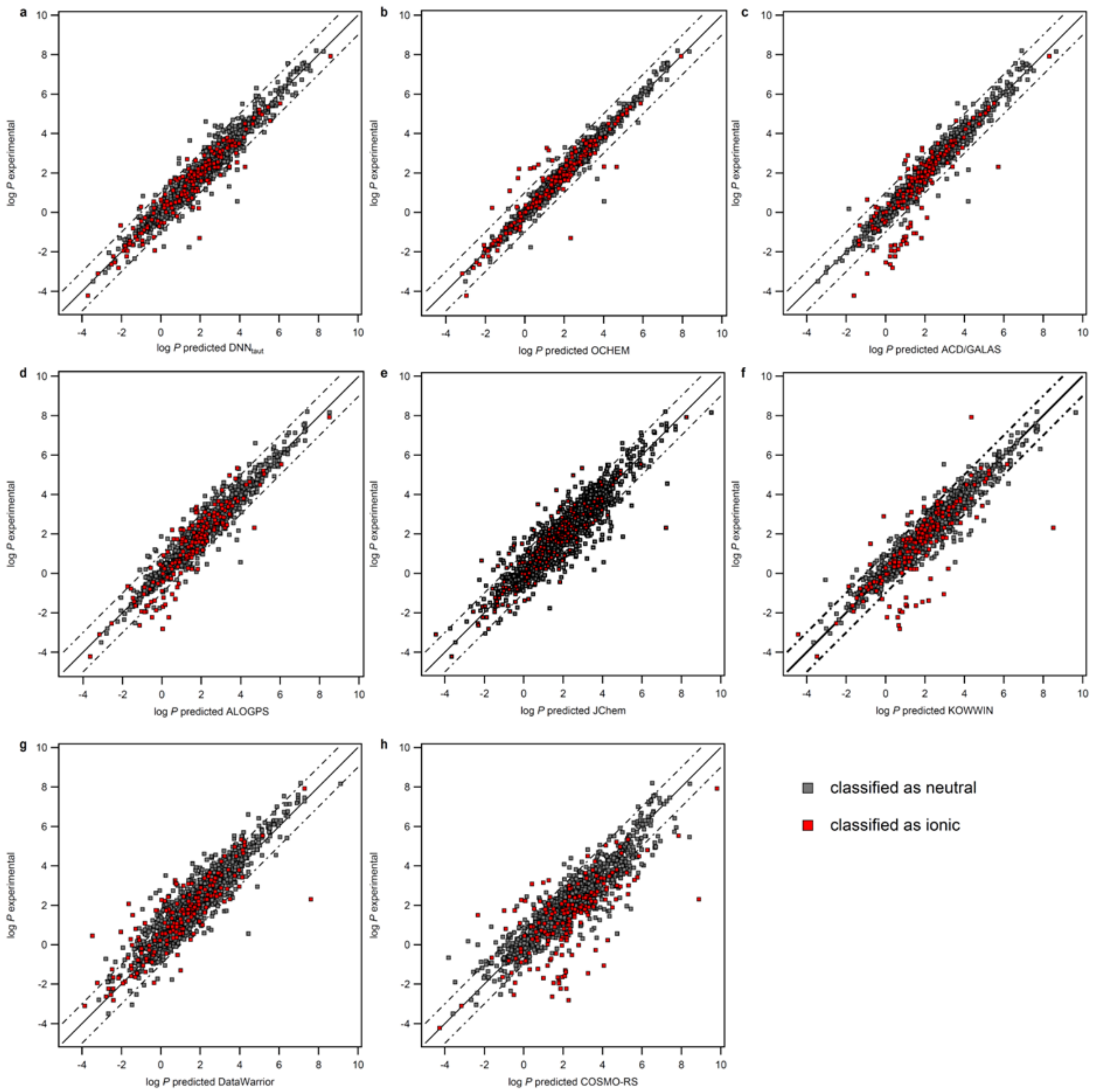

Supplementary Figure 1 Predictions of log *P* values for the test set by our DNN$_{taut}$ and 7 selected tools. The structure representation of the test set chemicals are based on the original SMILES codes. Neutral chemicals are marked in grey, potential ions are marked in red.

Supplementary Figure 2 – Comparison of the prediction performance for the test set of our DNN_{taut} to other tools based on randomly selected SMILES representations (including tautomers)

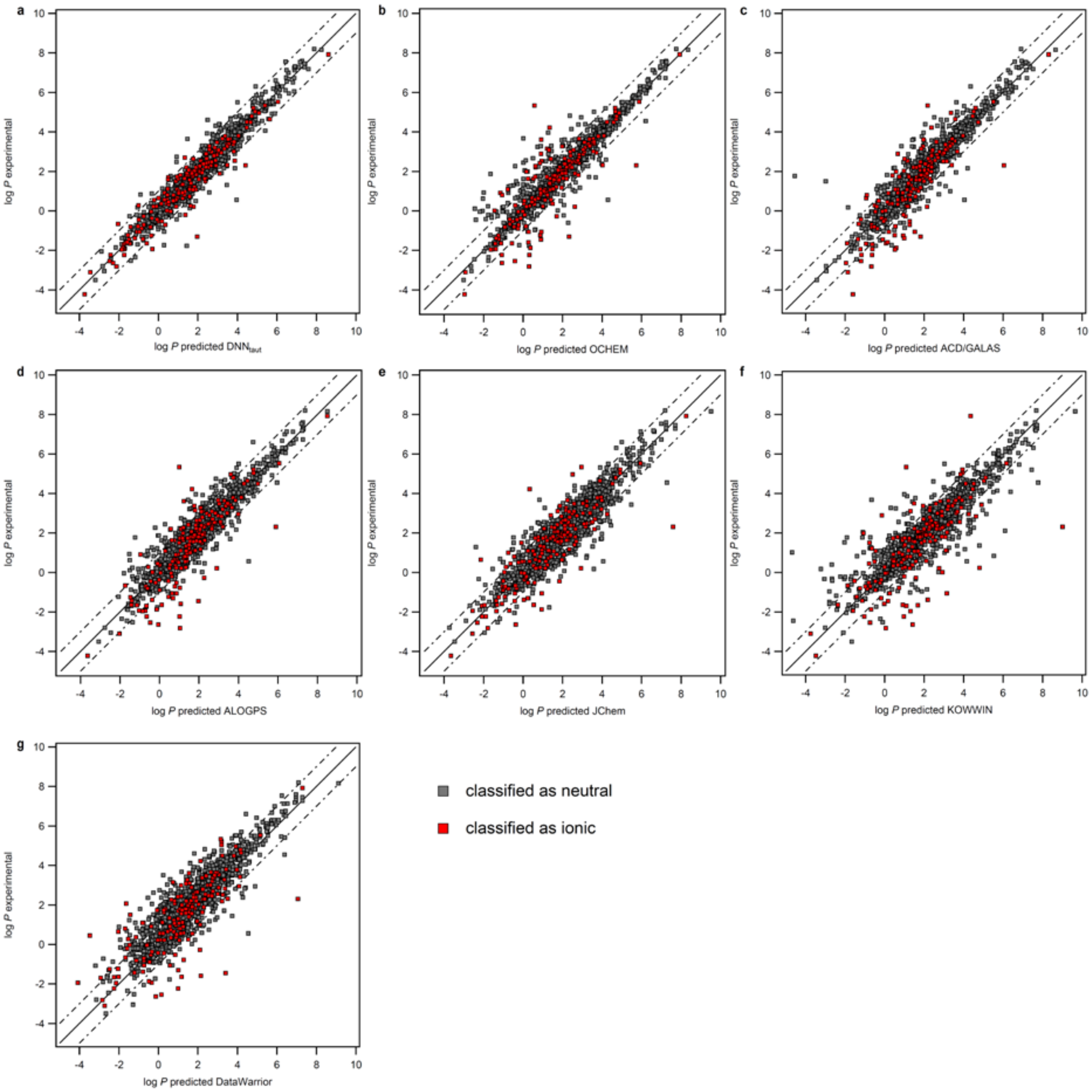

Supplementary Figure 2 Predictions of log *P* values for the test set by our DNN_{taut} and 6 selected tools (COSMO-RS is not included, because no tautomers were calculated). The structure representation of the test set chemicals was randomly selected (one SMILES per chemical) from SMILES codes (initial, canonical, with explicit Hs) including SMILES codes of all tautomers (multiple datapoints per chemical in case of tautomers). Neutral chemicals are marked in grey, potential ions are marked in red.

Supplementary Figure 3 – Characterization of the dataset of Martel et al..

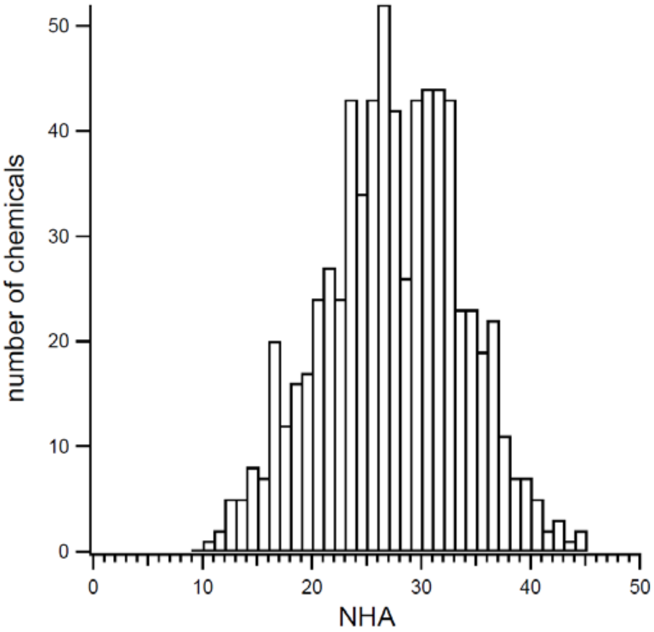

Supplementary Figure 3 The range of non-hydrogen atoms NHA reflecting the size of the molecules for the chemicals included in the dataset of Martel et al..

Supplementary Figure 4 – Predictions of the log P values from the dataset of Martel et al..

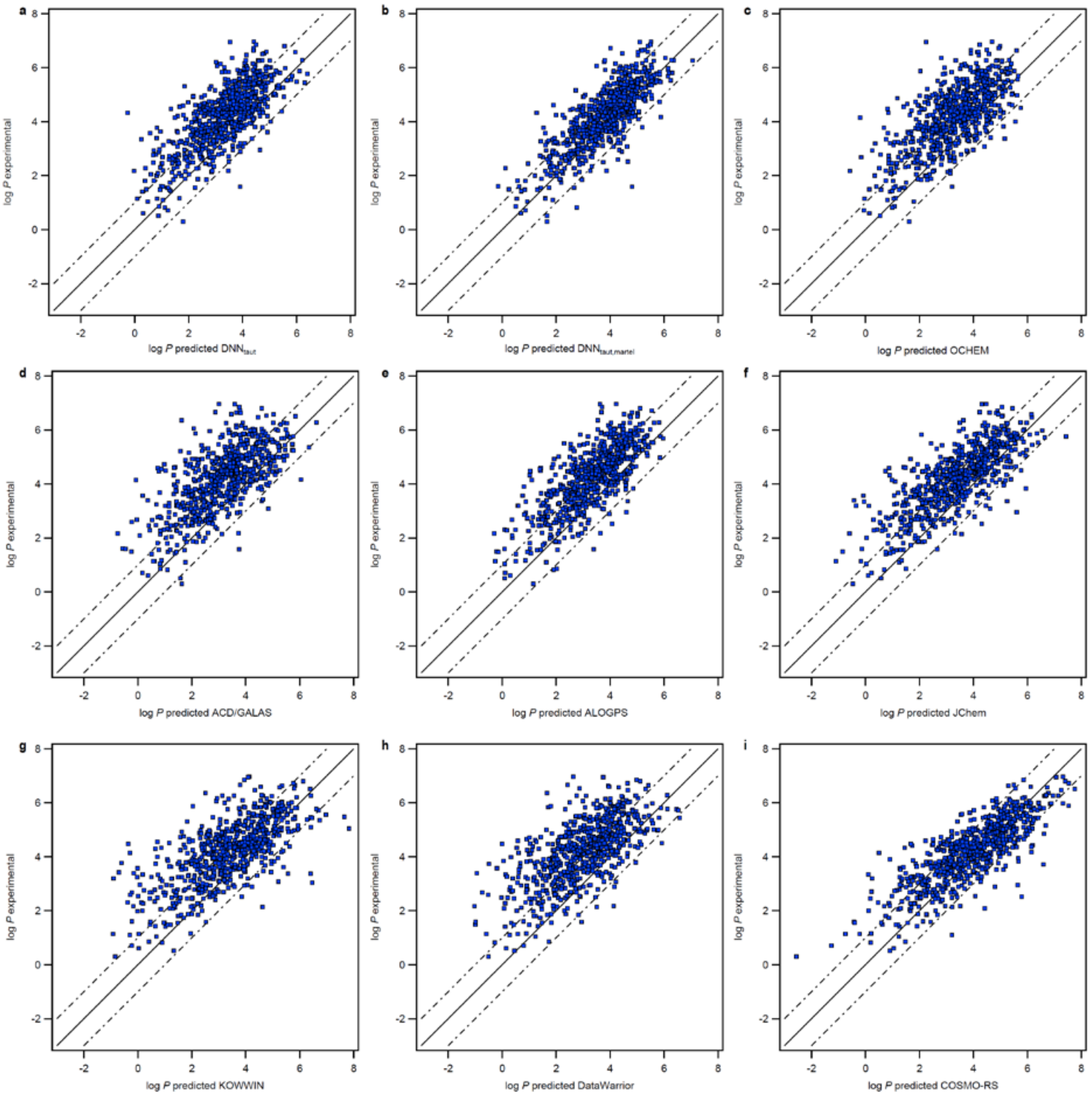

Supplementary Figure 4 Predictions of log *P* values for the dataset of Martel et al. by our DNN_{taut}, $DNN_{taut,martel}$, and 7 other tools.

Supplementary Figure 5 – Influence of the number of non-hydrogen atoms NHA on the prediction performance for the test set of our DNN$_{taut}$ to other tools based on the original SMILES

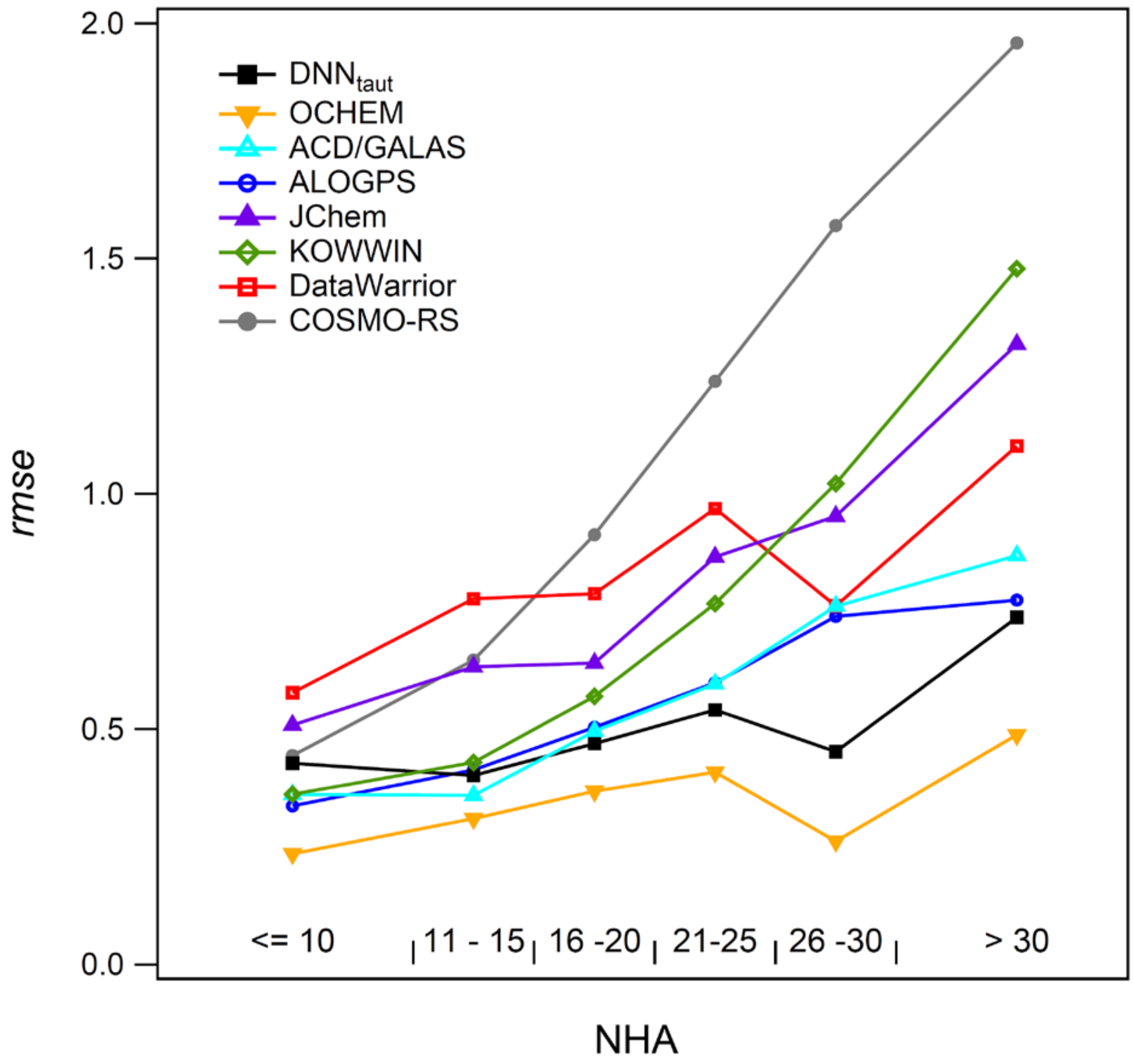

Supplementary Figure 5 Predictions of log *P* values for the test set (represented by the original SMILES) by our DNN$_{taut}$ and 7 selected tools. The *rmse* values are shown for the range of non-hydrogen atoms NHA reflecting the size of the molecules.

Supplementary Figure 6 – Influence of the number of non-hydrogen atoms NHA on the prediction performance for the test set of our DNN_{taut} to other tools based on the original SMILES without consideration of ionic chemicals

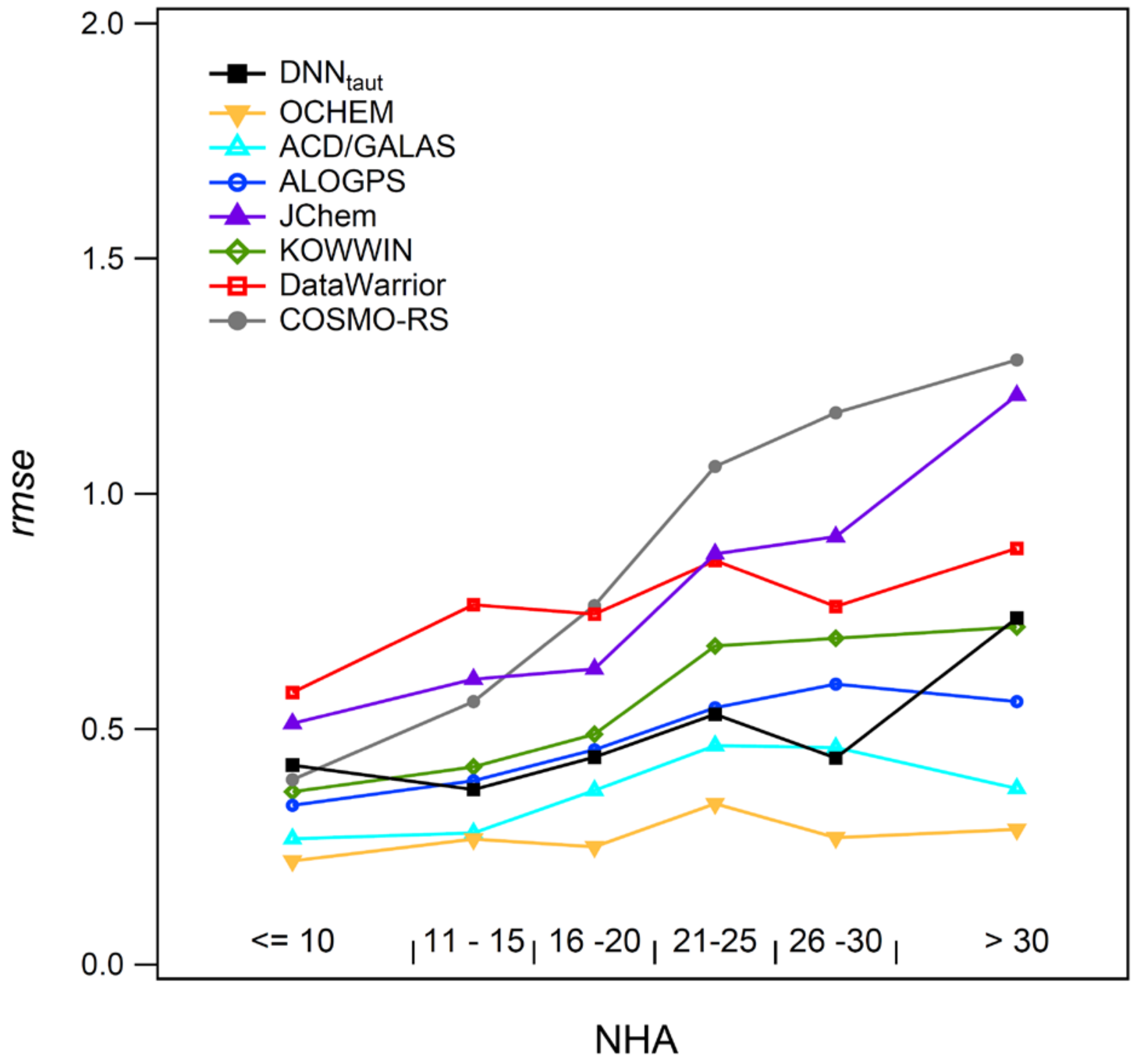

Supplementary Figure 6 Predictions of log *P* values for the test set (represented by original SMILES, only neutral chemicals included) by our DNN_{taut} and 7 selected tools. The *rmse* values are shown for the range of non-hydrogen atoms NHA reflecting the size of the molecules.

Supplementary Figure 7 – Rmse values of DNN_{taut} and DNN_{mono} depending on the number of epochs or training set size

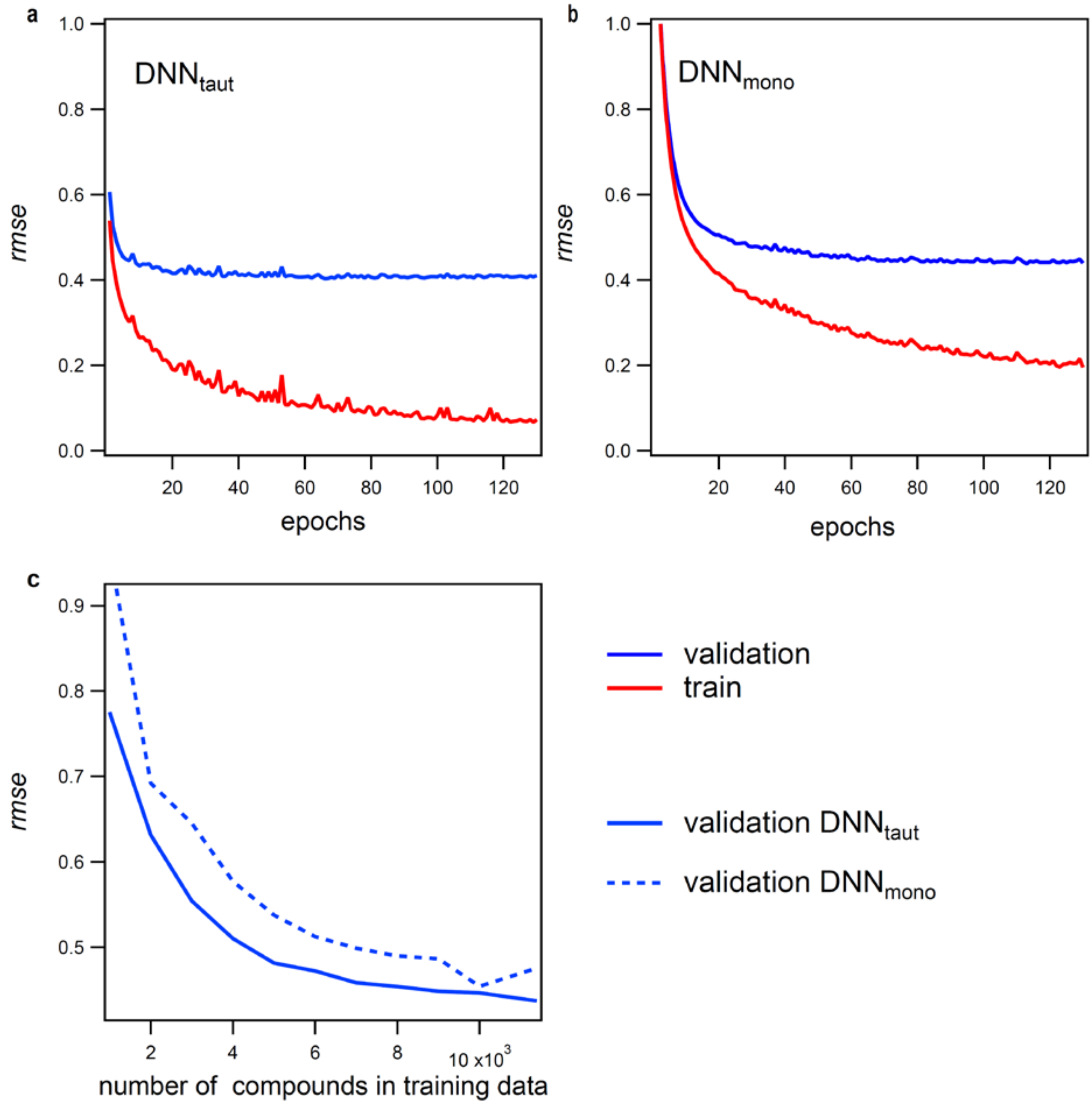

Supplementary Figure 7 *Rmse* values over the number of epochs for training set and validation set of DNN_{taut} (a) and DNN_{mono} (b), and the *rmse* values for the validation sets when the training size (number of chemicals) is stepwise increased (c).

Supplementary Table 1 – The different data sets can be composed of different SMILES representations (original, canonical, including explicit H atoms, tautomers), and may contain or exclude ions. Multiple SMILES representations per chemical may be present in the dataset, or alternatively one representation per chemical is selected.

SMILES data set	original SMILES	canonical SMILES	SMILES including explicit H atoms	tautomers	multiple SMILES representations per chemical	ions
all SMILES variants*	☑	☑	☑	☑	☑	☑
original SMILES**	☑					☑
original SMILES without ions	☑					
randomly selected***	☑	☑	☑	☑		☑
randomly selected *** without ions	☑	☑	☑	☑		
canonical SMILES		☑				☑
SMILES including explicit H atoms			☑			☑
SMILES of most likely tautomer	☑			☑		☑

*this SMILES data set is used as training set for the DNN_{taut}

** this SMILES data set is used as training set for the DNN_{mono}

***including tautomers

Supplementary Table 2 – Prediction results rmse of our DNN models and other prediction tools for various SMILES test sets. Mean value and variance were estimated using bootstrapping. Random sampling with replacement was used to generate N=1000 datasets per analyzed test set. If the rmse value of the original test set deviated from the calculated mean of the rmse distribution (N=1000; one rmse per dataset), the mean value was reported to symmetrize the confidence intervals. The variance was determined as the standard mean error.

	original SMILES		original SMILES without ions		SMILES of most likely tautomer[a]		SMILES randomly selected[b]		SMILES randomly selected[b] without ions		canonical SMILES		SMILES including explicit H atoms		all SMILES variants[c]	
model	*rmse*	*sdev*	*rmse*	*sdev*	*rmse*	*sdev*	*rmse*	*sdev*	*rmse*	*sdev*	*rmse*	*sdev*	*rmse*	*sdev*	*rmse*	*sdev*
DNN_{taut}	0.47	±0.02	0.45	±0.02	0.47	±0.02	0.47	±0.02	0.45	±0.02	0.47	±0.02	0.46	±0.02	0.46	±0.01
DNN_{mono}	0.50	±0.02	0.49	±0.02	0.54	±0.02	0.80	±0.03	0.72	±0.03	0.58	±0.04	1.01	±0.03	1.07	±0.01
COSMO-RS	0.97	±0.03	0.77	±0.03	-	-	-	-	-	-	-	-	-	-	-	-
DataWarrior	0.80	±0.02	0.75	±0.02	0.84	±0.02	0.92	±0.02	0.86	±0.02	0.80	±0.02	0.79	±0.02	1.16	±0.01
ACD/GALAS	0.50	±0.03	0.36	±0.02	0.54	±0.03	0.65	±0.03	0.58	±0.04	0.51	±0.03	0.50	±0.03	0.90	±0.01
JChem	0.72	±0.02	0.69	±0.02	0.75	±0.02	0.74	±0.03	0.70	±0.02	0.70	±0.02	0.69	±0.02	0.93	±0.02
KOWWIN	0.65	±0.04	0.51	±0.02	0.74	±0.04	0.92	±0.04	0.84	±0.04	0.74	±0.06	0.73	±0.06	1.37	±0.01
ALOGPS	0.50	±0.02	0.45	±0.02	0.56	±0.02	0.66	±0.03	0.58	±0.02	0.50	±0.02	0.50	±0.02	1.00	±0.01
OCHEM	0.34	±0.02	0.27	±0.02	0.48	±0.03	0.65	±0.03	0.57	±0.03	0.33	±0.02	0.33	±0.02	0.95	±0.01

[a]all tautomers were generated from the original SMILES using JChem, the most-likely tautomer was used

[b]including tautomers

[c]all SMILES representations for each chemical were used in the test set (multiple representations per chemical). In this case the *rmse* of the predictions is unbalanced due to chemicals which are represented by a large number of tautomer forms (we avoid to use this evaluation and prefer to use one randomly selected tautomer/SMILES variant instead).

Supplementary Table 3 – Prediction results of DNN_{taut} and other prediction tools for SAMPL6. Mean value was estimated using bootstrapping. Random sampling with replacement was used to generate N=1000 datasets per analyzed test set. If the rmse value of the original test set deviated from the calculated mean of the rmse distribution (N=1000; one rmse per dataset), the mean value was reported to symmetrize the confidence intervals.

SAMPL6 ID	log *P* exp	DNN_{taut}	DNN_{mono}	COSMO-RS	OCHEM	ACD/GALAS	KOWWIN	DataWarrior	ALOGPS	JChem
rmse		0.33	0.31	0.37	0.49	0.51	0.53	0.60	0.45	0.39
SM02	4.09	4.38	3.84	4.42	3.95	4.18	4.39	4.14	3.86	4.34
SM04	3.98	4.21	3.67	3.86	3.69	4.27	3.81	3.44	3.74	3.82
SM07	3.21	2.97	3.10	3.48	2.95	3.41	3.16	2.83	3.14	3.22
SM08	3.10	3.16	3.13	2.85	3.57	2.70	2.63	2.41	2.55	3.06
SM09	3.03	3.62	3.33	3.44	3.11	3.74	3.50	3.23	3.22	3.30
SM11	2.10	2.04	1.88	2.00	1.42	1.51	1.04	0.54	1.38	1.29
SM12	3.83	4.40	3.82	3.82	3.61	4.45	4.07	3.90	3.81	4.06
SM13	2.92	3.27	3.22	3.84	3.26	3.38	3.62	3.50	3.62	3.66
SM14	1.95	2.18	2.06	2.21	2.55	1.64	2.08	2.04	2.46	2.31
SM15	3.07	2.63	2.35	2.77	2.90	2.02	2.52	2.38	2.60	2.84
SM16	2.62	2.60	3.02	3.05	3.81	2.89	3.36	3.02	3.20	3.06

Supplementary Table 4 – Prediction results of DNN_{taut} and other prediction tools for the dataset of Martel et al.. Mean value and variance were estimated using bootstrapping. Random sampling with replacement was used to generate N=1000 datasets per analyzed test set. If the rmse value of the original test set deviated from the calculated mean of the rmse distribution (N=1000; one rmse per dataset), the mean value was reported to symmetrize the confidence intervals. The variance was determined as the standard mean error.

Martel	DNN_{taut}	$DNN_{taut,martel}$	DNN_{mono}	$DNN_{mono,martel}$	COSMO-RS	OCHEM	ACD/GALAS	KOWWIN	DataWarrior	ALOGPS	JChem
rmse	1.23	0.84	1.35	0.89	0.93	1.32	1.44	1.38	1.61	1.25	1.23
sdev	±0.03	±0.03	±0.03	±0.03	±0.03	±0.03	±0.04	±0.04	±0.04	±0.03	±0.03

References

1. Mansouri K, Grulke CM, Richard AM, Judson RS, Williams AJ. An automated curation procedure for addressing chemical errors and inconsistencies in public datasets used in QSAR modelling. SAR QSAR Environ Res 27, 939-965 (2016).

2. Martel S, et al. Large, chemically diverse dataset of log P measurements for benchmarking studies. Eur J Pharm Sci 48, 21-29 (2013).

3. Brooke DN, Dobbs AJ, Williams N. Octanol - Water Partition Coefficients (P) - Measurement, Estimation, and Interpretation, Particularly for Chemicals with P-Greater Than-10(5). Ecotoxicology and Environmental Safety 11, 251-260 (1986).
